# The Increasing Problem of Wound Bacterial Burden and Infection in Acute and Chronic Soft-Tissue Wounds Caused by Methicillin-Resistant *Staphylococcus aureus*

**Published:** 2007-11-16

**Authors:** Robert H. Demling, Barbara Waterhouse

**Affiliations:** Brigham and Women's Hospital, Burn and Trauma Center, Boston, MA.

## Abstract

Methicillin-resistant *Staphylococcus aureus* (MRSA) has become a leading cause of colonization and infection in both acute and chronic soft-tissue wounds. **Objective:** Our objective is to define this current epidemic problem caused by both community-acquired MRSA (CA-MRSA) and hospital-acquired MRSA (HA-MRSA), focusing on the similarities and differences between these 2 isolates as well as the impact on wound management decisions. **Methods:** Methods used include a literature review on the growth of the current MRSA problem and its International scope. In addition, a current up-to-date assessment had been made of the problem and the current approach to management of MRSA in acute soft-tissue and chronic wounds. Burns are not discussed because this injury usually does not fit either categories and is managed quite uniquely. **Results:** Results included the following: (1) There are very distinct properties of CA-MRSA and HA-MRSA, which must be considered for acute and chronic wound care. Management of both requires rigorous barrier precaution techniques to avoid cross-contamination. The presence of MRSA as a carrier state increases the risk of both a systemic and local wound infection in the carrier. There are large and increasing reservoirs of CA-MRSA and HA-MRSA worldwide leading to more bacteremias and wound problems. Topical antimicrobial therapy has not been addressed in managing MRSA in acute and chronic wounds. **Conclusion:** Conclusions include the fact that both HA-MRSA and CA-MRSA wound infections are rapidly increasing, especially with CA-MRSA. This high incidence requires appropriate wound prediction and management decisions as well as attempts to avoid further cross-contamination and reservoir growth. Topical antimicrobial therapy would seem to be an important component in controlling this tremendous problem. Yet this topic has yet to be adequately addressed.

The methicillin-resistant *Staphylococcus aureus* (MRSA) bacterium was recognized as an important clinical pathogen in the 1960s where it was found exclusively in a hospital setting.^1,2^

As recently as 20 years ago, MRSA was still considered to be a hospital-acquired pathogen. The usual infection was a bacteremia in a compromised patient on antibiotics after a prolonged hospital stay.

Today, MRSA is the leading cause of a nosocomial infection in most parts of the world. Also, MRSA is now the most identifiable cause of acute skin and soft-tissue infections seen in the urban emergency department setting.^3^–^6^

Over a relatively short-time period, MRSA has also evolved into 2 quite different types, each with different effects on wound epidemiology, and wound care strategy.

The initial variant of MRSA is now called health- or hospital-acquired methicillin resistant *Staphylococcus aureus* (HA-MRSA), as that is where the reservoir in large part resides.

A newer and very rapidly spreading type of MRSA has recently evolved in a community setting and in a healthy population and is referred to as community-acquired methicillin-resistant *Staphylococcus aureus* (CA-MRSA).

The CA-MRSA has been spreading at an epidemic rate and has now become a major cause of acute wound infection. The HA-MRSA is the type most likely to be seen in major burns. Both types of MRSA are seen in chronic wounds.

MRSA has become a huge worldwide health threat and an update on its current impact on wounds is warranted. The specific characteristics of HA-MRSA and CA-MRSA are presented, as well as, the impact these organisms have on acute and chronic wounds.^3^–^6^

## OVERVIEW OF HOSPITAL-ACQUIRED HA-MRSA AND CA-MRSA INFECTION

The first reports of MRSA infection occurred in the 1960s, felt to be from a widely disseminated “Iberian clone,” which was first seen in Europe.^1^,^2^ Clinical reports were confined to the hospital setting. The risk factors for an MRSA infection were noted to be those described in Table 1. The risk factors were confinement in a hospital unit, with high-risk patients, use of multiple antibiotics, and common use of intravenous catheters.

A number of epidemics were reported beginning in the 1970s, in various high-risk hospital settings (Table 2), with the major types of infections being bacteremias followed by pneumonias, and then wound infections.^7^–^10^

To date, bacteremias continue to be a much more common nosocomial infection problem with HA-MRSA.^2^,^11^,^12^ Recently, there has been a marked increase in the prevalence of MRSA throughout the world, especially in the past decade.^13^–^18^ The MRSA infections are tracked through causes of a bacteremia,^13^–^19^ most of which could be considered HA-MRSA due to the presence of risk factors.

Interestingly, the incidence of MRSA bacteremias varies dramatically throughout the world with only 0.5% of total cases of bacteremia reported in Iceland being MRSA, while 44% of the reported cases in Greece were caused by MRSA. There was an overall increase, in MRSA bacteremias, in Europe of 20%, between 1999 and 2002, with the increase in Germany and Italy being 50%.^15^–^24^ In Korea, more than 70% of nosocomial infection cases are caused by MRSA.^19^ The specific types of MRSA have been monitored carefully in the United States and Europe.

Around the year 1995, a new form of MRSA became evident, which was found outside the Health System.^23^–^28^ This organism, which apparently evolved from a different strain of MRSA than the hospital acquired was first identified in communities such as long-term care facilities. This *Staphylococcus aureus* named CA-MRSA has actually been defined on the basis of its differences from the health-acquired strain.

The clinical characteristics typically include no recent hospitalization or surgery, no antibiotics, no residence in a long-term care facility, no presence of any invasive medical devices, or no known infection with HA-MRSA (Table 3). To date, CA-MRSA infections tend to be disproportionately found in healthy children and young adults, sports participants, military recruits, and people with a lower socioeconomic status. Frequent skin-to-skin contact and hygiene problems are also common factors seen.

As opposed to bacteremia, typically seen with HA-MRSA, soft-tissue infections are very commonly caused by CA-MRSA.^29^–^30^

A number of microbiological differences have also been noted (Table 4).^29^–^31^

The CA-MRSA bacterium is more sensitive to antibiotics, with most isolates being sensitive to TMP/SMX, tetracycline, clindamycin, vancomycin, and linezolid.^28^–^32^ In addition, the USA 300 isolate appears to be the most common organism found in the United States. Also, the SCC mec type IV and the PVL are usually present, which are typically not found in HA-MRSA. The role of the PVL is not yet clear but it is known to correspond with tissue necrosis, a major feature seen in the soft-tissue infections.^28^–^32^

**Figure 1:**
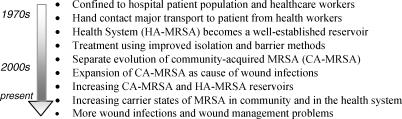
Evolution of MRSA and nosocomial infection from 1960 to present. From CDC research.

It appears that epidemiological efforts in the United State has clearly been focused on separating the 2 MRSA populations and their carrier states while on other parts of the world, the 2 populations are not separated (Fig 1).^15^–^24^

Like HA-MRSA, the spread of the CA-MRSA, in nosocomial infections, has been well recognized to be that of person-to-person contact. The only effective means of avoiding cross-containment has been very aggressive isolation techniques and improved hygiene, especially hand-washing vigilance.^4^

Surveillance cultures of hospitalized patients and care providers, with positive cultures, has been the common approach used to control the hospital problem. More recently, prophylactic treatment of all care providers and patients, in high-risk hospital units, has been shown to decrease MRSA infection in select series.^28^–^30^ A more aggressive approach known as “search and destroy” has been implemented in facilities in Europe, with high MRSA infection rates, using the rapid screening RDT testing, where positive patients and staff are treated.^31^ In the United States, intensive personnel screening has been shown to be effective but has not been implemented as Standard of Care. Topical intranasal Mupirocin cream is the most commonly used treatment for MRSA nasal colonization.^1^,^34^

Despite increasing vigilance, the prevalence of both forms of MRSA continues to increase. A MRSA carrier state has long been recognized in the healthcare system and is now much more evident in the community. The nares remains the most common site followed by the skin for HA-MRSA, while skin is the more common carrier site for CA-MRSA.^34^–^38^ Of importance with CA-MRSA is that the asymptomatic carrier also frequently develops an acute skin or a soft-tissue infection,^34^–^38^ while the hospital worker carrying HA-MRSA in the nares seldom becomes infected with the organism

As of 2001, the Centers for Disease Control and Prevention (CDC) estimated that there were an estimated 2 million MRSA carriers in the United States alone.^28^,^30^ This number has certainly increased since that time. Most of the MRSA carriers are now in the community, as opposed to 20 years ago when the hospital worker was the major reservoir.

Control of CA-MRSA has focused on hygiene education and avoiding the development of MRSA reservoirs, for example, in outpatient type facilities, through screening and prevention campaigns. Certainly prevention of the spread of MRSA from a known carrier, by proper barrier controls and education, is essential. Prophylactic use of mupirocin ointment in long-term care facilities has shown a decrease in infections. However there remains concern over the development of resistance. There was actually evidence of increasing mupirocin resistance beginning back in the 1970s with over usage.^38^38–40 Alternative preventive measures and treatments will need to be developed, probably using silver products, which are known to kill MRSA.^4^(M2)

## ACUTE SKIN AND SOFT-TISSUE INFECTION CAUSED BY MRSA EXCLUDING BURNS

The focus of this discussion is the management of typical traumatic soft-tissue wounds and/or dermatosis that becomes infected, with MRSA, and requires local wound care. These wounds are typically not exposed to the risk factors for HA-MRSA. Burn injury is a very distinct wound and will be described separately.

*Staphylococcus aureus* has always been a major source of infection in acute soft-tissue wounds, but MRSA has only been an infecting organism in a small fraction of the total. However, CA-MRSA has now rapidly become both a virulent, and extremely common wound pathogen.^42^–^49^

**Figure 2:**
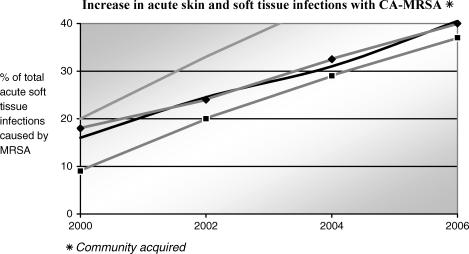
The percentage of total of acute soft tissue wounds, infected with MRSA, is shown over time for 4 recent emergency department based clinical studies.^21^,^32^,^43^,^44^

Currently, CA-MRSA is reported to be the most identifiable cause of acute skin and soft-tissue infections in urban emergency departments in the United States, with the incidence doubling in the last 3 to 4 years (Fig 2). In addition, the bacterial isolate involved is quite specific, with 97% of strains being the USA 300 type, and the majority also having the PVL. This gene may explain the typical tissue necrosis and abscess formation seen with this form of infection.

In general, infections caused by methicillin sensitive *Staphylococcus aureus* and methicillin resistant *Staphylococcus aureus* are quite similar. However, CA-MRSA tends to produce more abscess formation and tissue necrosis.

**Figure 3:**
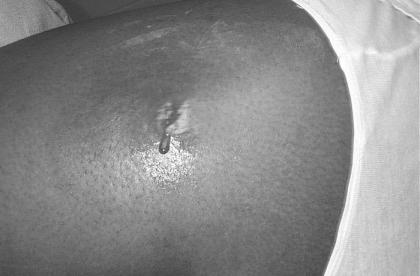
Necrotic, draining wound infected by CA-MRSA(“Spider Bite’). Note the purulent nature of the small wound with surrounding cellulitis.

**Figure 4:**
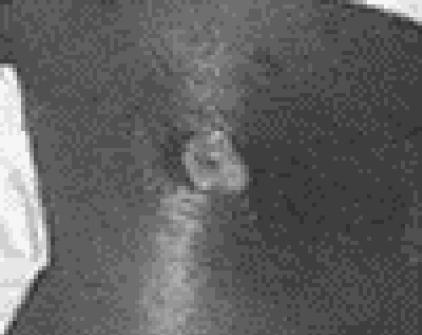
Necrotic pustule infected with CA-MRSA. Note the necrotizing property in the wound with surrounding cellulitis.

One common and quite interesting presentation of a CA-MRSA infection is that of a small painful skin pustule with surrounding cellulitis, often described as a “spider bite” which then rapidly evolves into an abscess with progressive cellulitis (Figs. 3 and 4).

The clinical profile of CA-MRSA infection is described in Table 3. More severe infections such as necrotizing fascitis are also seen especially in children and young adults, who tend to have the more severe soft-tissue infections with this organism.

The majority of patients, in the most recent multi center studies with acute skin and soft-tissue infections, were treated with a prophylactic B-lactam antibiotics not effective against CA-MRSA.^43^–^44^ Lack of effectiveness was detected later with the return of routine culture results. Interestingly, the majority of patients who had abscesses, which were initially drained, improved even with ineffective antibiotics, indicating the importance of aggressive local wound care. Only patients with inadequate drainage or significant cellulitis progressed before changing the antibiotic regime. 43,44

More recently, a rapid screening test RDT for MRS A has become available in most major centers. This technique uses a quick multiplex immunocapture-coupled PCR and only takes a few hours.

Because of the rapid increase in CA-MRSA acute infections, the CDC recently published new *Guidelines for the Management of Acute Skin and Soft Tissue Infections* (Table 5).

There are a number of highlighted approaches for acute wound management.

First of all CA-MRSA is likely to be the cause of an acute purulent soft tissue infection of the skin. Second, cultures should be obtained not only for specific patient wound-care decisions, but to develop a regional profile for this increasing pathogen. At present, there is not sufficient information available to recommend the need for specific typing and characterization of the MRSA. However, at some point in time, a genetic profile may be quite valuable in tracking a current cluster. Third, it appears that the CA-MRSA causes local tissue necrosis and aggressive unroofing of the wound leads to optimum results.

Fourth, empiric antibiotics are selected on the basis of evidence of local wound invasion with systemic symptoms. However, if there is a high regional incidence of CA-MRSA or risk factors for this infection are increased, appropriate CA-MRSA antibiotics should be selected (Table 6).

Community acquired MRSA is quite antibiotic sensitive.

Fifth, as previously mentioned, the acute care setting needs to follow rigorous infection control precautions, to avoid healthcare worker and environment colonization and subsequent increased risk of cross-contamination.

A point of controversy may be the approach to the carrier states in personnel and patients. The typical carrier sites are the nares and the skin. It is well known that a carrier state can lead to both a soft tissue infection in the same patient and to cross-contamination.^35^,^37^–^45^ At present, there is insufficient data for acute wound care facilities, to recommend prophylactic decolonization of the healthcare staff and patients.

Finally, patient education becomes a key factor in management to avoid spreading CA-MRSA to an entire household, chronic care facility, sports team, or any other contacts of the patient.

Of major interest is the apparent lack of attention given to the approach of local wound care for the acute MRSA-infected wound. It would seem logical that a rapid local control of the organism using a tested appropriate topical antimicrobial agent would decrease wound bacterial load and also diminish the potential for cross-contamination. Local wound care management, in the literature reviewed, appears to be at the discretion of the healthcare provider. This approach is very different from that used in the management of burns where topical agents are considered standard of care.

Burns will not be discussed in this review because although most burns are acute soft-tissue wounds, usually all of the literature focuses on major burns in burn centers. Therefore, the impact of MRSA is only available for a very small portion of these wounds and the major problems are not related to the wounds but rather bacteremias and lung infections.

## CHRONIC WOUNDS AND MRSA

There is now a well-recognized increase in MRSA colonization and infection in chronic wounds.^52^–^57^ The increase appears to be comparable to the worldwide increase in MRSA in acute wounds. The MRSA presents two problems, the first relates to the chronic wound being a source of other MRSA nosocomial infections and the second relates to the impact of MRSA on the chronic wound itself.

It has been well demonstrated that patients admitted for an acute injury or illness, who have chronic wounds growing MRSA, have an increased risk of a bacteremia with MRSA being the organism.^57^

As previously described, the mortality of a, MRSA bacteremia is significantly higher than a methicillin-sensitive *Staphylococcus aureus*.77 The increasing reservoir of MRSA in the chronic wound population becomes a source of other nosocomial infections.^58^,^59^ The chronicity of the chronic wound also increase the risk of cross-contamination.

The best data on the effect of MRSA on the wound itself are in the diabetic ulcer population.^58^–^60^ *Staphylococcus aureus* appears to be the most common pathogen, among the gram-positive bacteria, isolated from the diabetic ulcer. Several recent reports have indicated an incidence of MRSA growth of up to 50% in these ulcers and the MRSA ulcers are more likely to become infected.^59^59–62 Suggesting increased virulence, CA-MRSA appears to be more virulent in a wound than HA-MRSA.

Infection in these studies was diagnosed according to the criteria proposed by the International Consensus on the Diabetic Foot.^60^ Interestingly, several studies have reported that there appears to be no difference in diabetic ulcer healing rate when colonized by MSSA, methicillin-susceptible *Staphylococcus auerus*, or MRSA.^58^–^61^ Of course, it is much more difficult to treat a multi-drug-resistant MRSA with systemic antibiotics that must be considered when discussing morbidity.^60^

Other studies have reported an increase in hospital stay, increased cost, and increased morbidity and mortality with an MRSA foot infection compared to other diabetic foot ulcer infections.^62^–^64^

Interestingly, many of these studies have indicated that the typical risk factors for the development of HA-MRSA are not prevalent today in this diabetic ulcer population, indicating that CA-MRSA is likely to be the pathogen of concern.^55^,^56^ Compared to the research on acute supportive wounds, research on chronic wounds has yet to distinguish MRSA isolates as to more specific microbiological features. However, a common theme is that effective control of the spread of MRSA in the diabetic ulcer population comes from knowledge of the likely pathogens in one's wound practice, including antibiotic sensitivity profiles, when systemic antibiotics are required.^65^,^67^

Adequate off-loading, wound debridement, optimum blood flow, and appropriate systemic antibiotics are the standard care for an infected diabetic ulcer, or one which has a heavy bacterial burden, no matter what the organism.^67^

There is very little data on the role or the type of topical antimicrobial agents to be used in an MRSA diabetic ulcer infection. Silver products in the form of foams, alginates, and controlled release dressings are quite popular.^69^ No other topical agent has been demonstrated to be effective in this patient population. However, the overall role of silver in controlling the increasing MRSA reservoir has not been addressed.

The only other chronic wound, which has been discussed in the literature, relative to this topic is the chronic venous stasis ulcer.^56^ Silver products are typically the topical agents most commonly discussed in the literature.^70^–^74^

It appears that MRSA colonization and infection are clearly increasing in the chronic wound population. The distinction between CA-MRSA and HA-MRSA is not clearly defined especially compared to the acute soft tissue wound. However, most data suggest that CA-MRSA is the predominate isolate. The presence of MRSA places the patient at risk for other more severe MRSA-related complications. In addition, at least for the diabetic ulcer, there appears to be a greater risk of colonization evolving to infection.^56^,^58^

The basic treatment approach to the increased bacterial bioburden or infected chronic wound is active debridement and systemic antibiotics. The role of topical antimicrobial agents in managing the growing problem of MRSA and the chronic wound remains poorly defined. At present, silver-based agents and dressings remain the most popular approach.

## SUMMARY

MRSA is now a major cause of wound infections and bacteremias worldwide, and this problem is growing at an epidemic pace.

There are currently 2 types of MRSA. There is the well-established hospital or hospital-acquired HA-MRSA, seen on the compromised patient population on antibiotics, especially with an intravascular device in place. More recently CA-MRSA has been established, typically seen in the young healthy, nonhospitalized population.

The multiantibiotic resistant HA-MRSA, typically transmitted by hand-to-skin contact, is an increasing cause of bacteremia worldwide. The relatively antibiotic sensitive CA-MRSA, also transferred by skin-to-skin contact, is an increasing cause of soft tissue infection. Both are common causes of wound colonization and bacterial burden.

The worldwide resistance of both types of MRSA reservoirs is increasing in both active infections and asymptomatic carrier states. The carrier states can be very dangerous leading to both bacteremias and other nosocomial infections in the carrier.

The impact of MRSA in acute and chronic soft-tissue wounds is described.

CA-MRSA is rapidly becoming the most common cause of acute soft-tissue wound infections. The carrier state of CA-MRSA is also rapidly expanding, increasing the risk of infections even in relatively minor wounds. The CDC's recent guidelines have reported that CA-MRSA must be considered to be the cause of any acute soft tissue infection and appropriate systemic antibiotic choices must be made.

In addition, rigorous isolation and barrier approaches, as well as patient-care education, need to be initiated to avoid further cross-contamination. Interestingly, there is really no guidelines described as to the appropriate approach to topical antibiotic therapy, although silver products are known to be effective against all variants of MRSA.

As to the chronic wound population, there is a clear increase in colonization, bacterial burden, and infection caused by MRSA, best documented in the diabetic ulcer population. Of importance is the fact that the chronic wound population with MRSA is at increased risk for both wound infections and systemic infections, especially bacteremias, if another acute illness occurs requiring hospitalization. These data reflect the dangers of MRSA in the chronic wound as a focus for more life-threatening nosocomial infections.

The distinction between CA-MRSA and HA-MRSA is not as well described for the chronic wound as for the acute wound. However, there does appear to be clear indication that CA-MRSA predominates in the outpatient population.

As with the acute wound, there is little definitive data as to the appropriate approach to topical therapy for the colonized or infected chronic wound. Silver release products are most common since MRSA is sensitive to silver.

It would be logical that topical therapy would be an important component in the management of all of the types of wounds described, to decrease the reservoirs of both types of MRSA.

## Figures and Tables

**Table 1:** Risk factors for HA-MRSA*

Long hospital presence in ICU/burn/NICU	Prior or present history of antibiotic use	In-dwelling intravenous catheter	Previous history of MRSA	Immune impaired or compromised Host	Open wounds

*HA-MRSA indicates health-acquired hospital-acquired methicillin resistant *Staphylococcus aureus*; ICU, intensive care unit; NICU, neonatal intensive care unit; and MRSA, methicillin-resistant *Staphylococcus aureus*.

**Table 2:** Common populations for HA-MRSA epidemics and the common infection site.*

	
Burn centers	Neonatal units/NICU
Bacteremia	Bacteremia
Wounds	Lungs
Lungs	Renal dialysis centers
Bone marrow transplant	Bacteremia
Bacteremia	Long-term care
Lungs	Pneumonia
Wounds	Chronic wounds
ICU medical/surgical	
Bacteremia	
Lungs	
Wounds	

*HA-MRSA indicates health environment or hospital acquired; NICU, neonatal intensive care unit; and ICU, intensive care unit.

**Table 3:** Clinical profile with CA-MRSA infections*,†


Commonly seen in healthy children, young adults
Sports, military recruits, skin-to-skin contact
Low socioeconomic status
Hygiene problems
No criteria suggestive of HA-MRSA
Primary infection of skin and soft tissues
Abscess formation

*Not seen with HA-MRSA.

†CA-MRSA indicates community-acquired methicillin-resistant *Staphylococcus aureus*; HA-MRSA, hospital-acquired Methicillin-resistant *Staphylococcus aureus*.

**Table 4:** Microbiological properties of CA-MRSA not seen with HA-MRSA


Antimicrobial sensitivity
Presence of SCC mec type gene
Presence of Panton-Valentine leukocidin toxin gene

**Table 5:** CDC guidelines for management of acute SSTIs.*

	
1. Consider CA-MRSA a likely cause of acute SSTIs including purulent “Spider Bites”	4. Utilize Empiric Antibiotic Therapy based on wound indications considering CA-MRSA coverage based on risk factors
2. Obtain wound cultures and sensitivity For individual patient purposes To determine local CA-MRSA characteristics	5. Maintain Standard Infection Control Precautions in the Treatment area. MRSA can be readily transmitted in the health care environment
3. Utilize aggressive incision and drainage approaches to the wound, followed by standard wound management practices†	6. Patient Infection Control Education is important to avoid the spread of CA-MRSA back into the community

*CDC indicates Centers for Disease Control and Prevention; SSTIs, skin and soft-tissue infections; and CA-MRSA, community-acquired methicillin-resistant *Staphylococcus aureus*.

†Currently there are no good data as to the ideal topical antimicrobial therapy for MRSA skin and soft tissue infections.

**Table 6:** Antimicrobial therapy for suspected community-acquired methicillin-resistant Staphylococcus aureus wound infection.*


Suspected infection: Doxycycline, bacterium, tetracyline, clindamycin†
Serious infection/bacteria: Vancomycin or linezolid^48^,^49^
Topical antibiotic: mupirocin, silver products^51^,^52^

*The standard management of a typical hospital-acquired methicillin-resistant *Staphylococcus aureus* infection would be Linezolid or Vancomycin.

†Clindamycin resistance is an increasing problem.
